# Supplementary material for: Features of Highly Homologous T-Cell Receptor Repertoire in the Immune Response to Mutations in Immunogenic Epitopes
Source: Int J Mol Sci. 2024 Nov 23;25(23):12591. doi: 10.3390/ijms252312591 (PMC11641755; doi:10.3390/ijms252312591)
Supplement: Supplementary file 1 [file ijms-25-12591-s001.zip › Suppl fig 3 TCR repertoires of immunogenic epitopes from HLA-A_24_02.pdf]

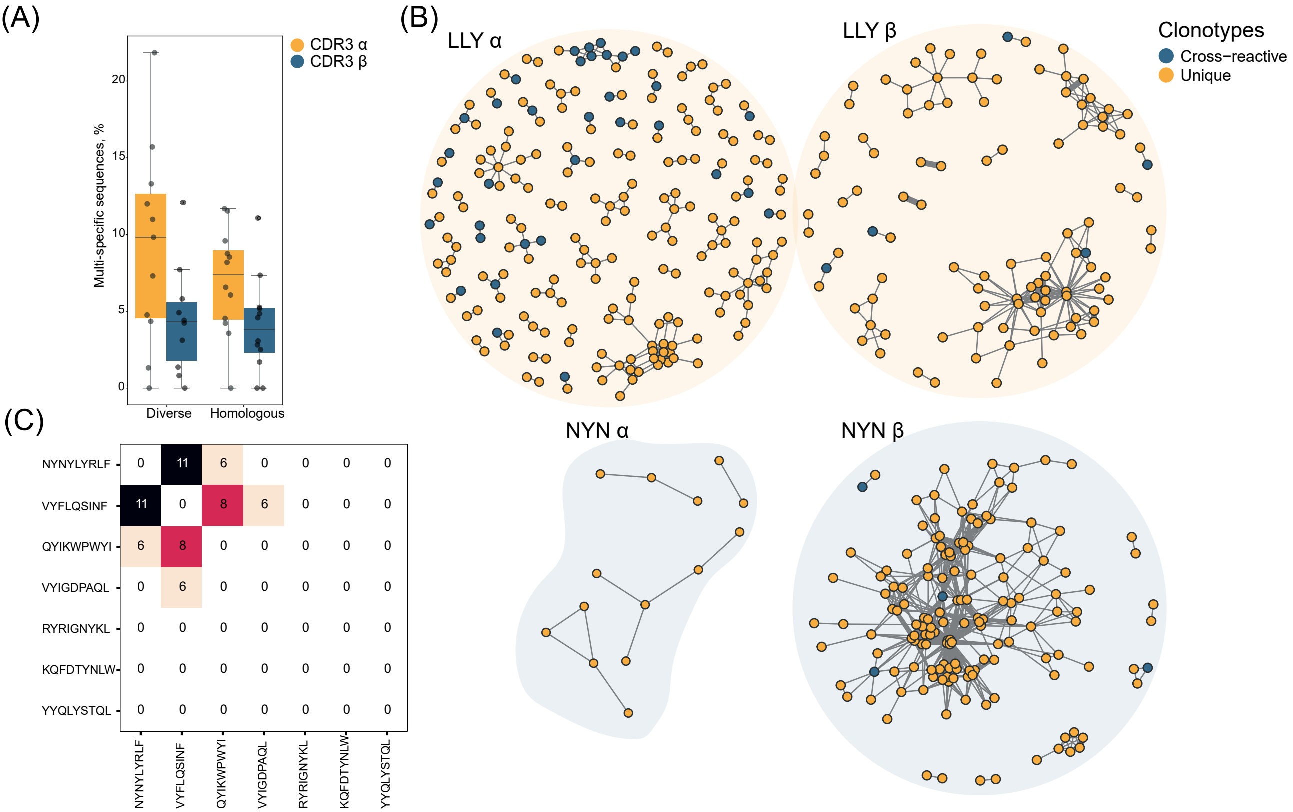
(A)
CDR3 α
CDR3 β
Multi-specific sequences, %
0
5
10
15
20
Diverse
Homologous
(B)
LLY α
LLY β
NYN α
NYN β
Clonotypes
Cross−reactive
Unique
(C)
NYNYLYRLF 0 11 6 0 0 0 0
VYFLQSINF 11 0 8 6 0 0 0
QYIKWPWYI 6 8 0 0 0 0 0
VYIGDPAQL 0 6 0 0 0 0 0
RYRIGNYKL 0 0 0 0 0 0 0
KQFDTYNLW 0 0 0 0 0 0 0
YYQLYSTQL 0 0 0 0 0 0 0
NYNYLYRLF
VYFLQSINF
QYIKWPWYI
VYIGDPAQL
RYRIGNYKL
KQFDTYNLW
YYQLYSTQL
