# Supplementary material for: Features of Highly Homologous T-Cell Receptor Repertoire in the Immune Response to Mutations in Immunogenic Epitopes
Source: Int J Mol Sci. 2024 Nov 23;25(23):12591. doi: 10.3390/ijms252312591 (PMC11641755; doi:10.3390/ijms252312591)
Supplement: Supplementary file 1 [file ijms-25-12591-s001.zip › Supplementary Figures description.pdf]

Supplementary Figure S1. (**a**) SARS-CoV-2 structure, and full-length SARS-CoV-2 spike (S) protein. Close-up view of epitope residues (blue); (**b**) SARS-CoV-2 impairs the immune response either by disrupting antigen presentation via MHC-I to CD8+ T cells (upper part) or by preventing T cell activation through the TCR (lower part). Once the virus enters epithelial cells via endocytosis (1), its genomic RNA is released to synthesize viral proteins using the host cell's protein expression machinery (2). Recognized as non-self molecules, viral proteins are lysed into peptides, which are then loaded onto MHC-I molecules and presented to CD8+ CTLs, thereby activating the cytotoxic response (3). However, due to mutations in specific antigen peptides (e.g., spike protein-derived antigens), these peptides may lose their binding affinity to HLA-I molecules. As a result, the peptides are either not loaded or are loaded unstably onto the corresponding HLA molecules (4). This leads to reduced or absent CD8+ T cell activation due to low affinity or lack of HLA-I-peptide recognition by the TCR (5). Alternatively, if mutations do not result in a loss of binding affinity, the peptides are loaded onto the corresponding HLA molecules (6) but fail to be recognized by T cells specific to the wild-type peptides (7); (**c**) Molecular bases of epitope–HLA-I complexes. Interactions between NYN peptide and HLA-A*24:02 (from PDB:7F4W); (**d**) Molecular bases of TCR–epitope–HLA-I complexes. Overview of TCR-NYN- HLA-A*24:02 complex (8YE4). Close-up view of interactions of epitope residues (blue) with TCRα (light green) and TCRβ (dark green). For all the structures, HLA heavy chains are yellow, the NYN peptides is blue.

Supplementary Figure S2. (**a**) Clusters of CDR3α and/or CDR3β sequences of epitope-specific CD8+ T cells. Each node represents a unique CDR3β amino-acid sequence or public sequences, the node size is proportional to the number of identical clonotypes. Lines connect similar sequences, with Hamming distance = 1. Colors indicate source. Only clusters with two or more members are shown; (**b**) Comparison of immune response to mutated epitopes between repertoires with low and high homology of TSRs. Mann–Whitney p value is shown. Data from the current study was included; (**c**) V- and J-gene usage in the main NYN-specific cluster.

Supplementary Figure S3. (**a**) The percentage of multi-specific sequences in diverse and homologous repertoires; (**b**) Clusters of LLY- and NYN-specific sequences. Each node represents a unique amino-acid sequence. Lines connect similar sequences, with Hamming distance = 1. Blue color indicates multi-specific sequences; (**c**) Overlap of CDR3β sequences between immunogenic epitopes from HLA-A*24:02.

Supplementary Figure S4. Gating strategy: (**a**) selecting cells, (**b**) selecting single cells, (**c**) selecting CD8+ cells, that corresponds to the Jurkat E6-1 TPR cells expressing the epitope-specific TCRs, (**d**) selecting GFP+ cells, that corresponds to the activated cells after peptide stimulation, (**e**) negative control GFP+ gating, (**f**) positive control GFP+ gating.
