# Supplementary figures and images for: Features of Highly Homologous T-Cell Receptor Repertoire in the Immune Response to Mutations in Immunogenic Epitopes
Source: Int J Mol Sci. 2024 Nov 23;25(23):12591. doi: 10.3390/ijms252312591 (PMC11641755; doi:10.3390/ijms252312591)

(A)

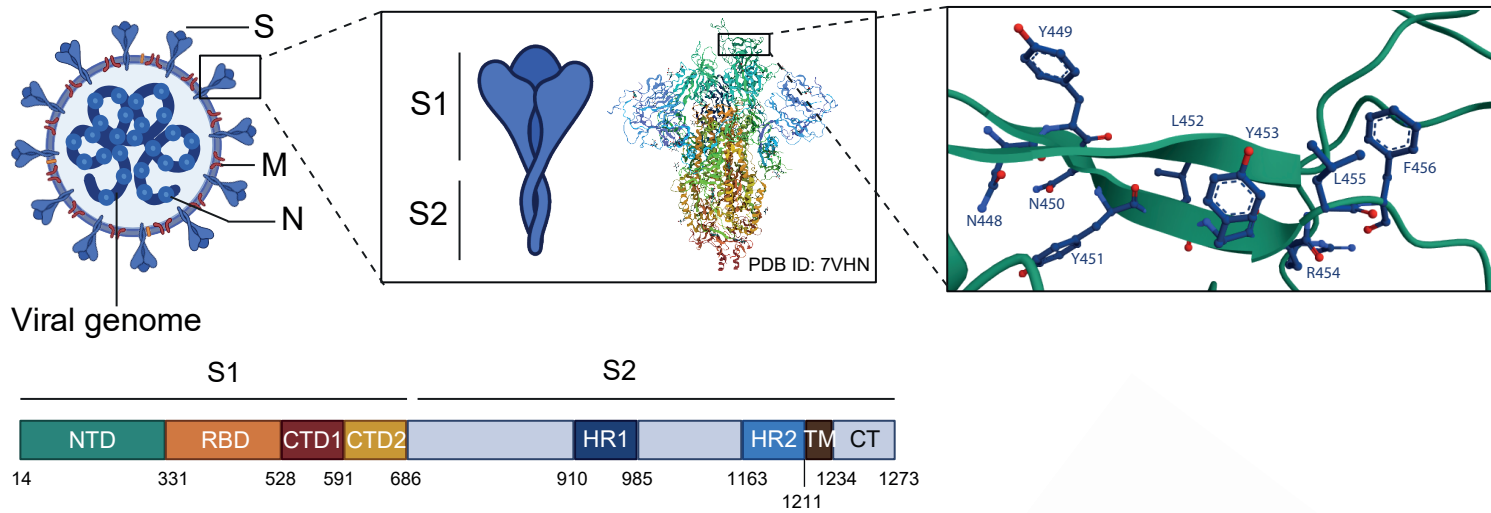

(B)

### 1 Impaired antigen presentation by MHC-I

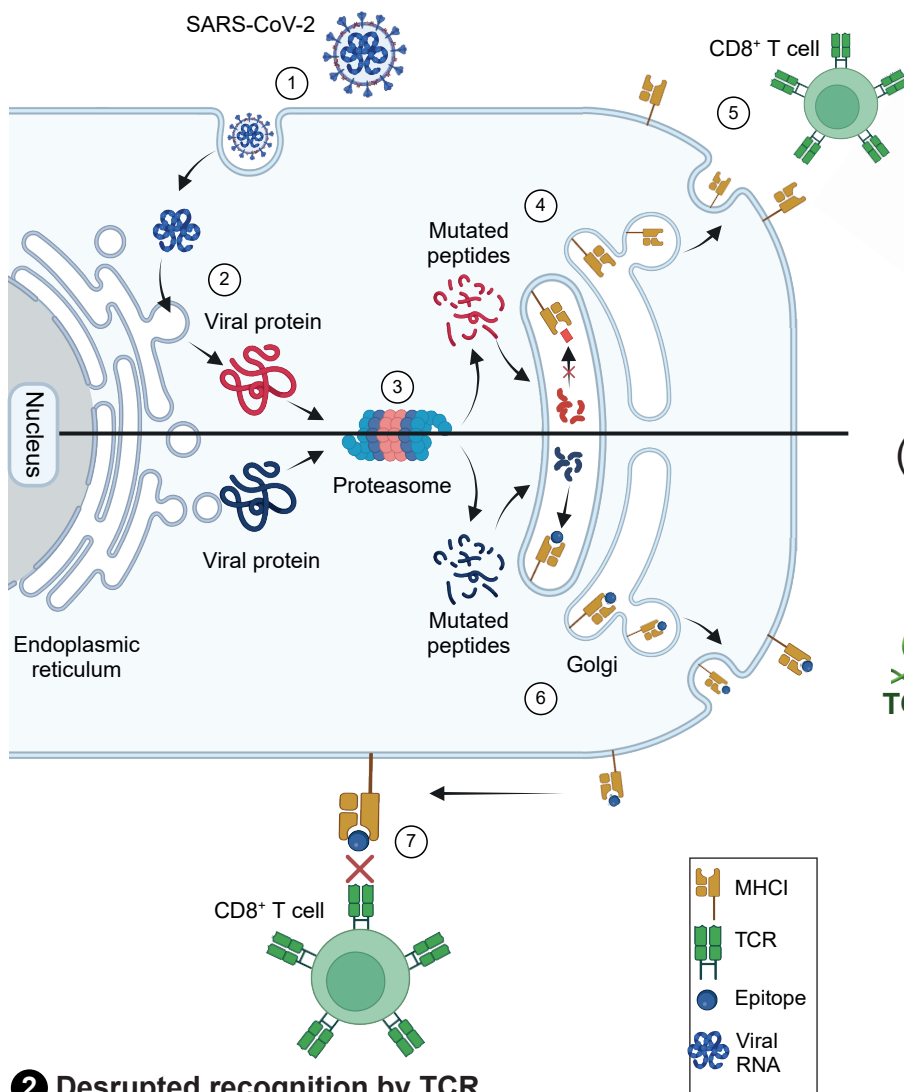

### 2 Desrupted recognition by TCR

(C)

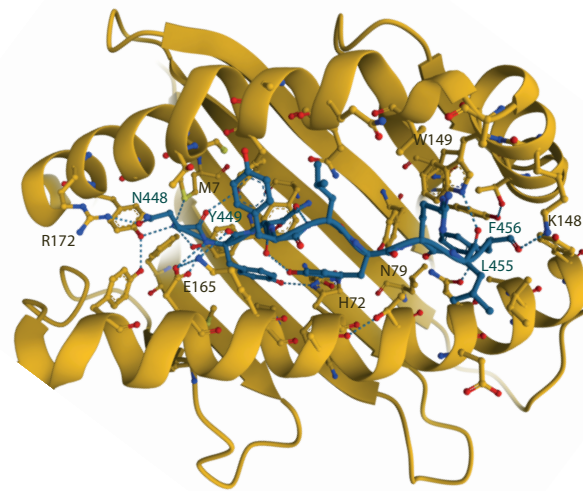

(D)

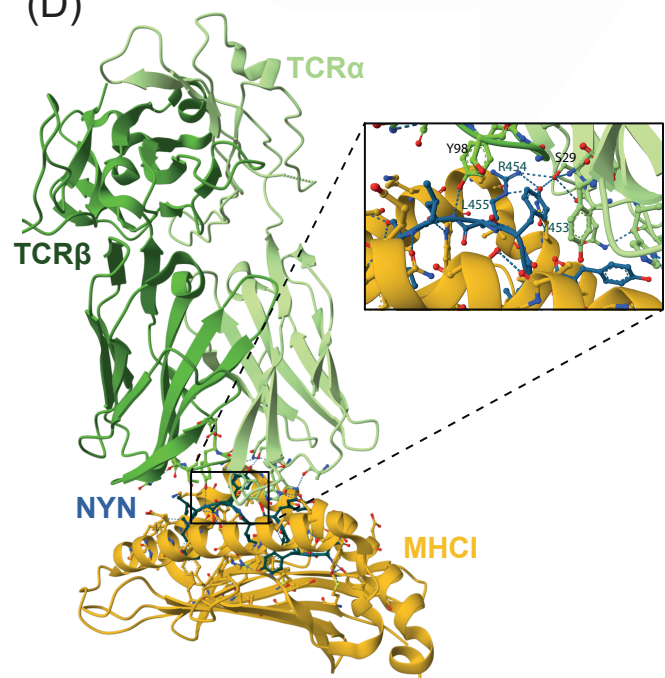

Supplement: Supplementary file 1 [file ijms-25-12591-s001.zip › Suppl fig 1 SARS-CoV-2 structure and mechanisms of immune evasion.pdf]

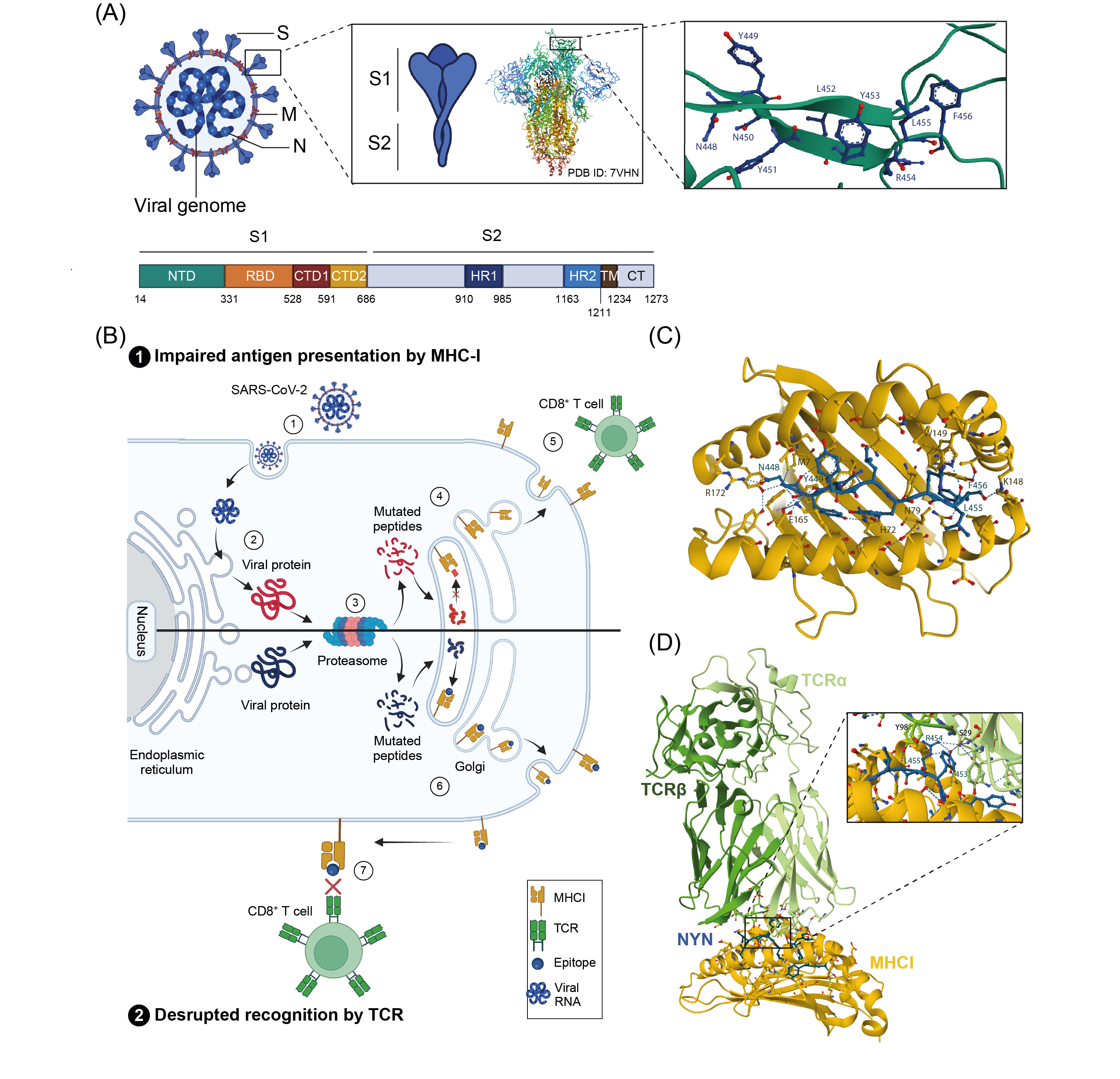

Supplement: Supplementary file 1 [file ijms-25-12591-s001.zip › Suppl fig 1 SARS-CoV-2 structure and mechanisms of immune evasion.png]

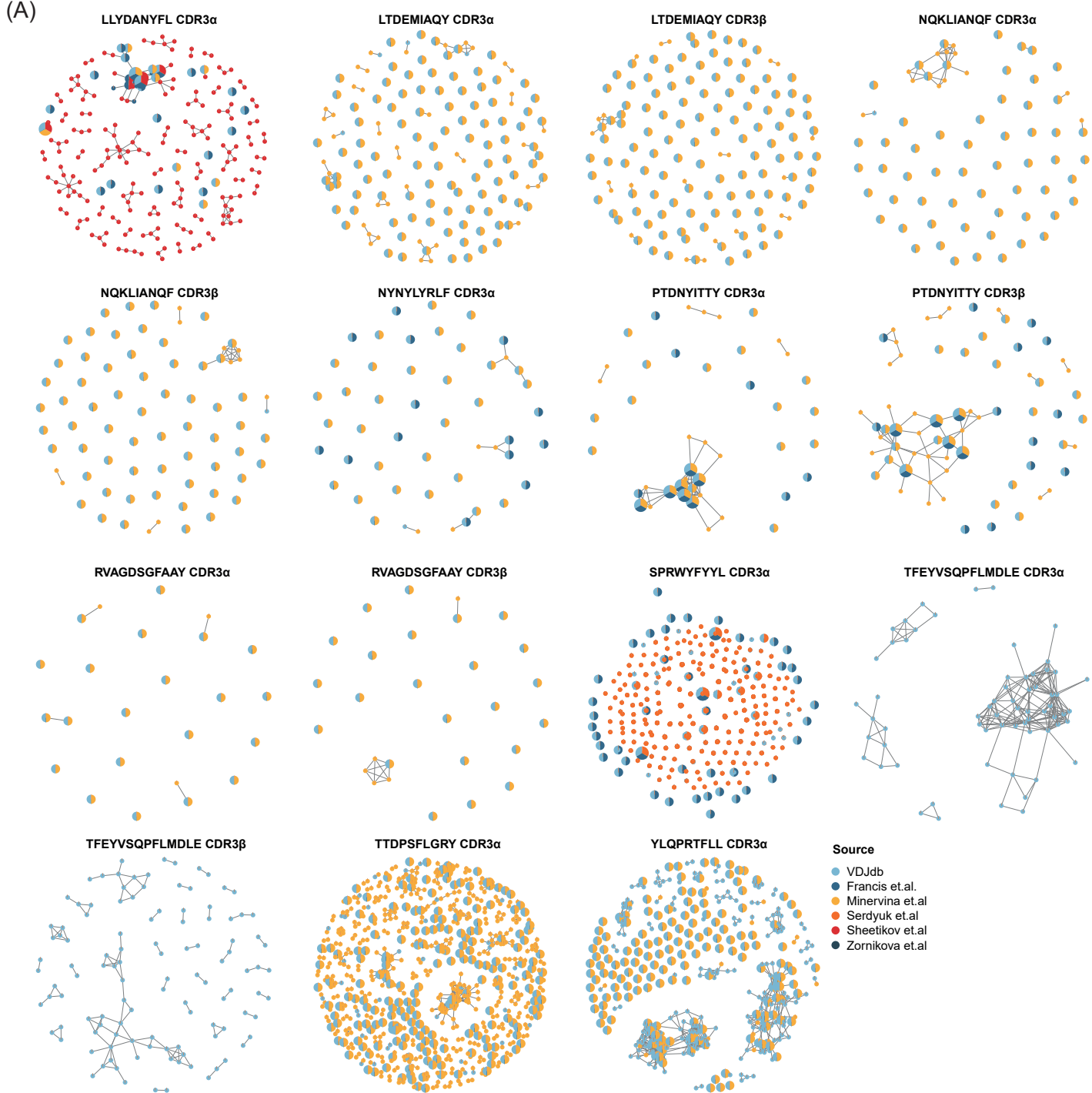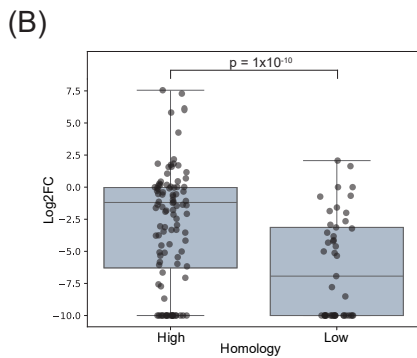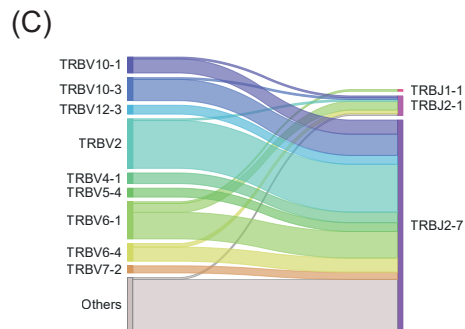

Supplement: Supplementary file 1 [file ijms-25-12591-s001.zip › Suppl fig 2 The cluster structures for various epitopes from HLA-A_24_02.pdf]

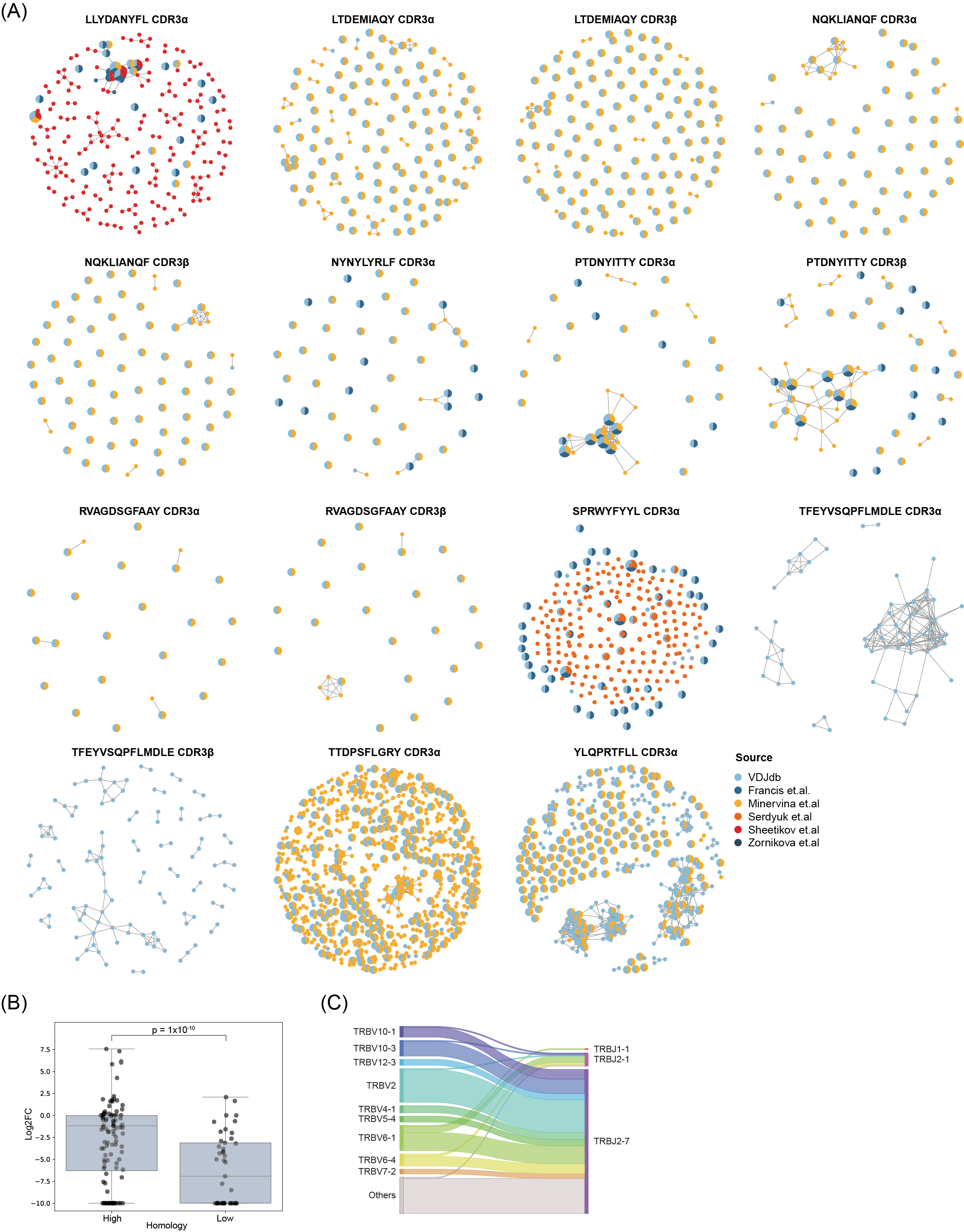

Supplement: Supplementary file 1 [file ijms-25-12591-s001.zip › Suppl fig 2 The cluster structures for various epitopes from HLA-A_24_02.png]

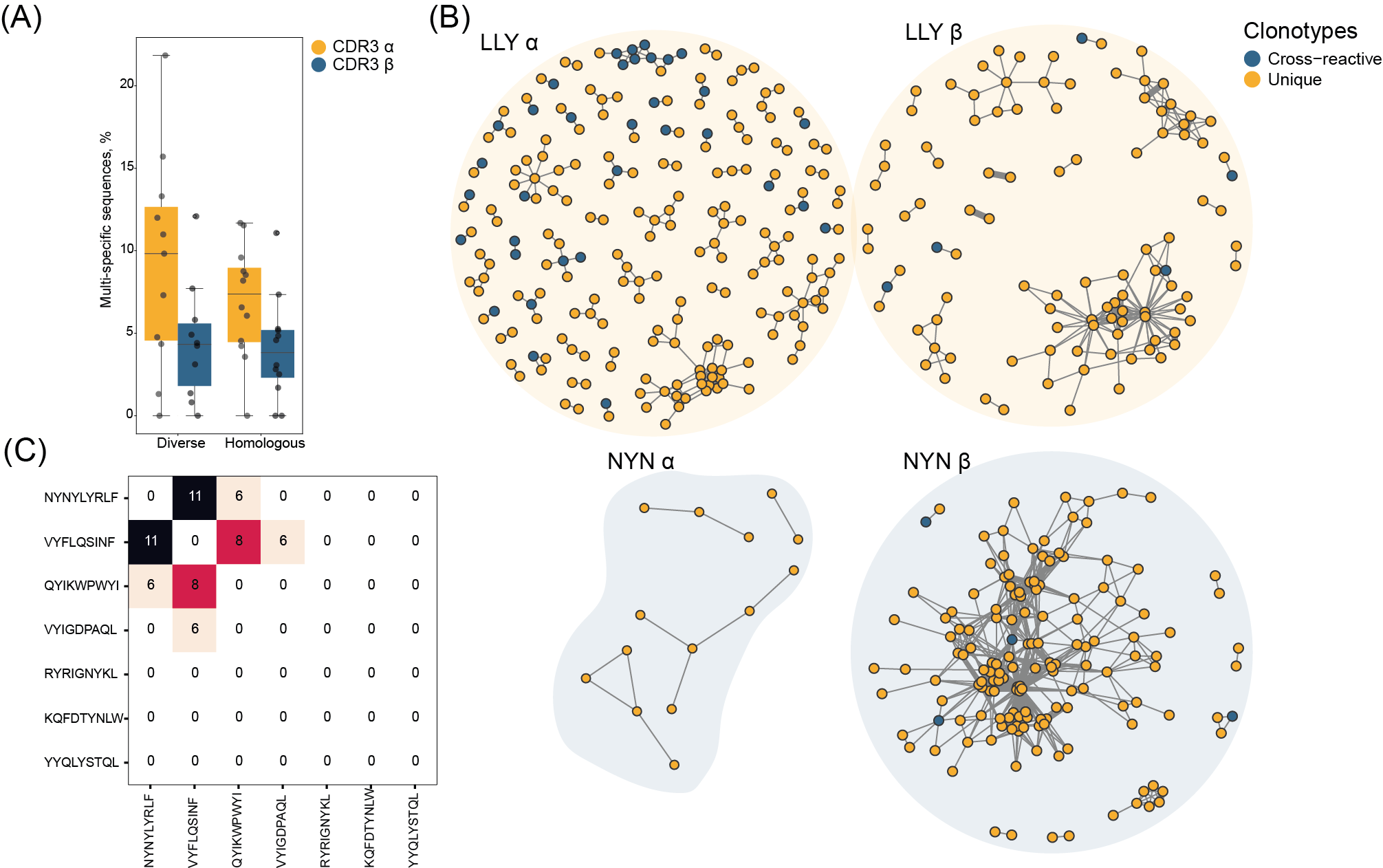

Supplement: Supplementary file 1 [file ijms-25-12591-s001.zip › Suppl fig 3 TCR repertoires of immunogenic epitopes from HLA-A_24_02.png]

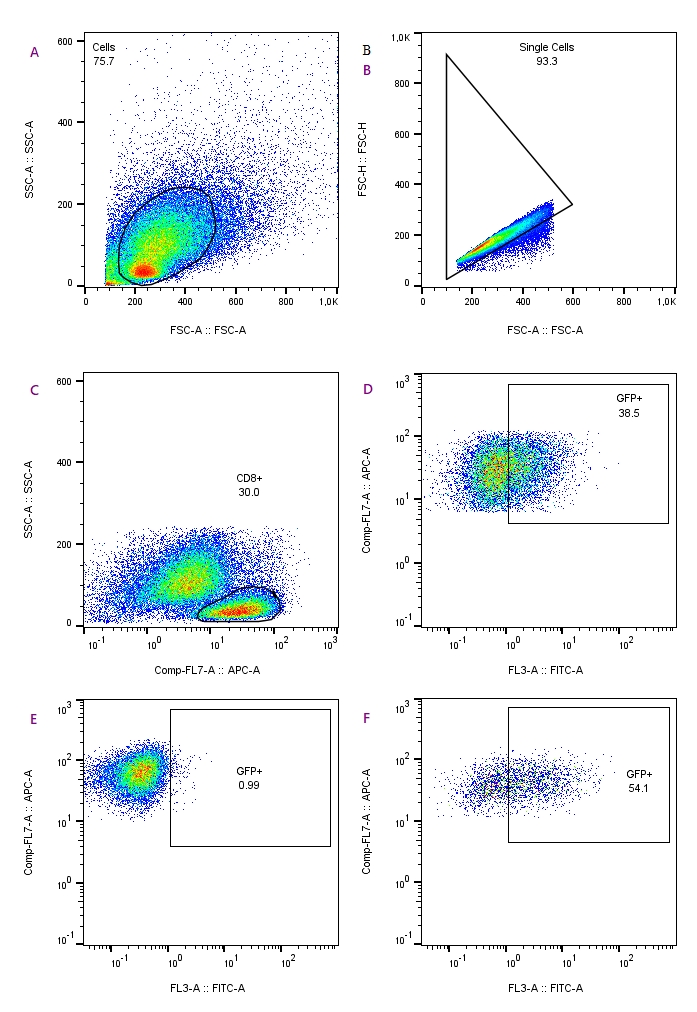

Supplement: Supplementary file 1 [file ijms-25-12591-s001.zip › Suppl Fig 4 Gating strategy.jpg]

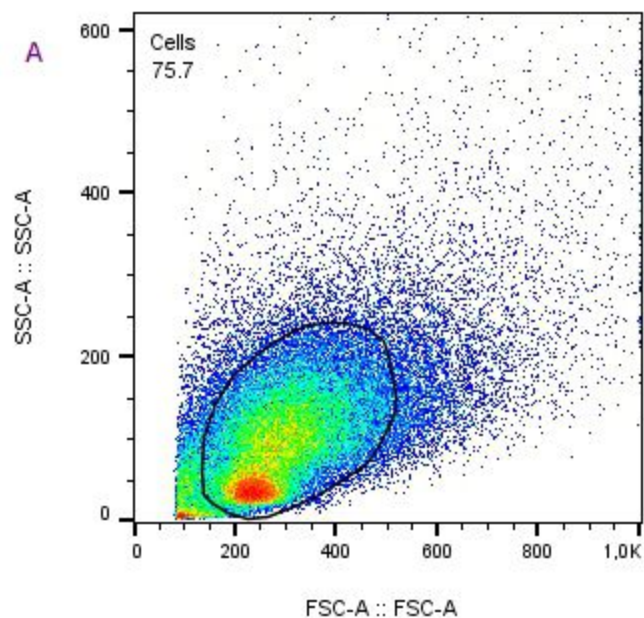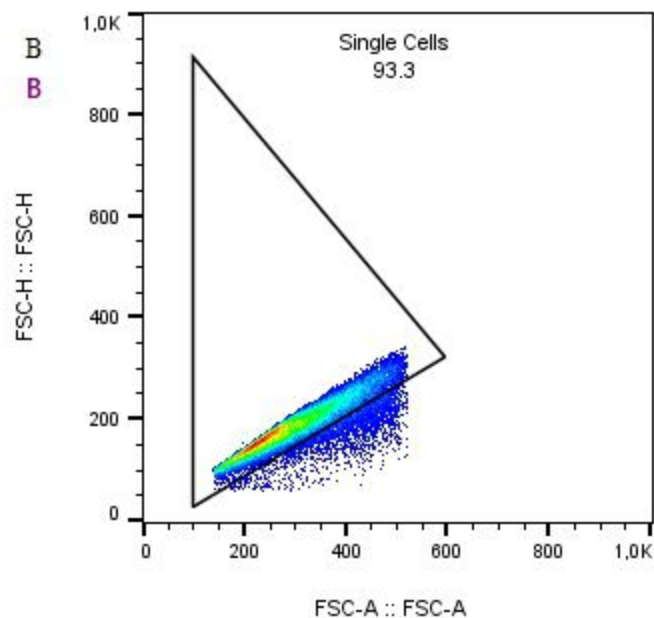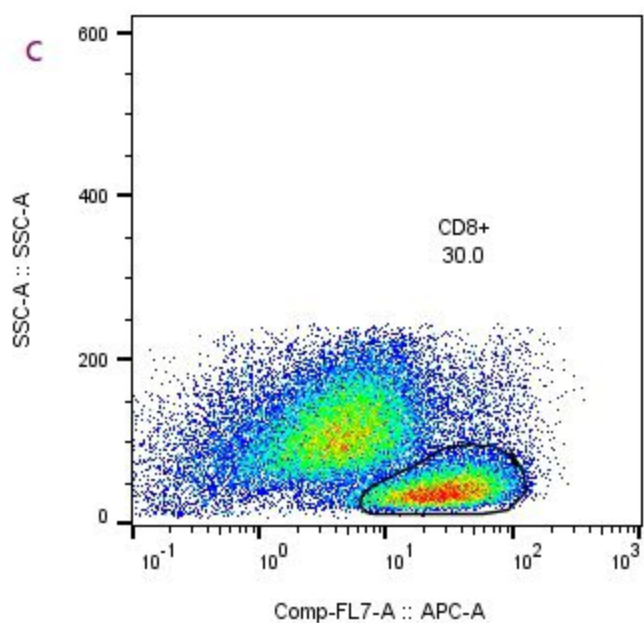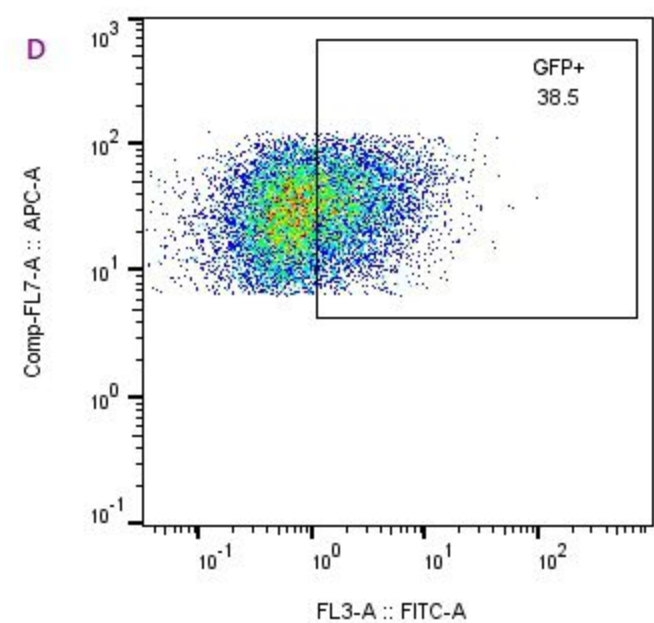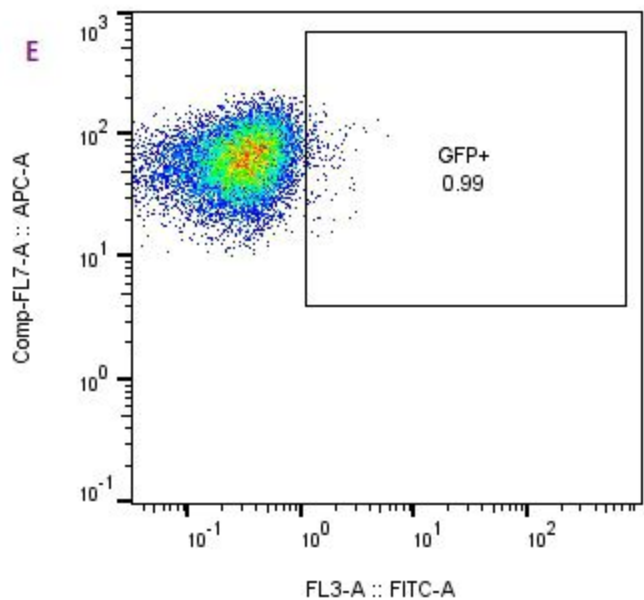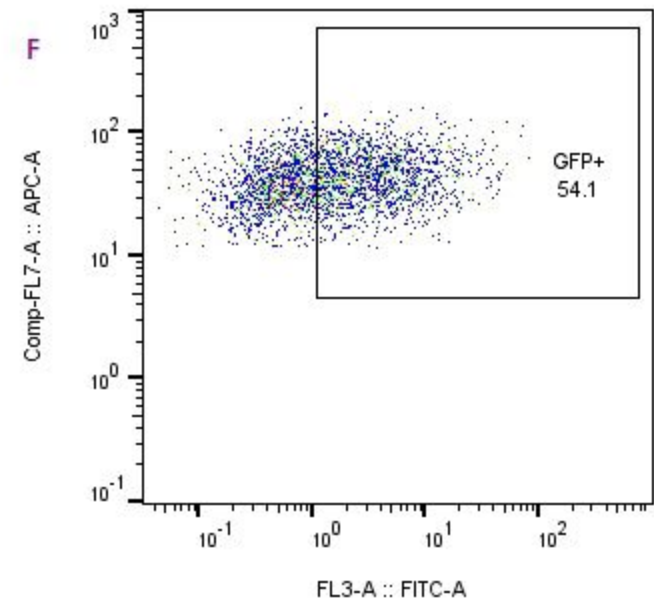

Supplement: Supplementary file 1 [file ijms-25-12591-s001.zip › Suppl Fig 4 Gating strategy.pdf]
